# The Simrad EK60 echosounder dataset from the Malaspina circumnavigation

**DOI:** 10.1038/s41597-021-01038-y

**Published:** 2021-10-01

**Authors:** Xabier Irigoien, Thor Klevjer, Udane Martinez, Guillermo Boyra, Anders Røstad, Astrid C. Wittmann, Carlos M. Duarte, Stein Kaartvedt, Andrew S. Brierley, Roland Proud

**Affiliations:** 1 AZTI - BRTA, Herrera Kaia, Portualdea z/g – 20110, Pasaia, Gipuzkoa Spain; 2 IKERBASQUE, Basque Foundation for Science, Bilbao, Spain; 3 Institute of Marine Research, PO Box 1870 Nordnes, 5817 Bergen, Norway; 4 King Abdullah University of Science and Technology (KAUST), Red Sea Research Center, Thuwal, 23955-6900 Saudi Arabia; 5 MARUM - Center for Marine Environmental Sciences University of Bremen FVG-Ost Leobener Strasse 2, 28359 Bremen, Germany; 6 Department of Biosciences, University of Oslo, PO Box 1066 Blindern, 0316 Oslo, Norway; 7 School of Biology, Scottish Oceans Institute, Gatty Marine Laboratory, University of St Andrews, East Sands, St Andrews, Fife, KY16 8LB, Scotland UK

**Keywords:** Marine biology, Biooceanography

## Abstract

We provide the raw acoustic data collected from the R/V *Hesperides* during the global Malaspina 2010 Spanish Circumnavigation Expedition (14th December 2010, Cádiz-14th July 2011, Cartagena) using a Simrad EK60 scientific echosounder operating at 38 and 120 kHz. The cruise was divided into seven legs: leg 1 (14th December 2010, Cádiz-13th January 2011, Rio de Janeiro), leg 2 (17th January 2011, Rio de Janeiro-6th February 2011, Cape Town), leg 3 (11th February 2011, Cape Town-13th March 2011, Perth), leg 4 (17th March 2011, Perth-30th March 2011, Sydney), leg 5 (16th April 2011, Auckland-8th May 2011, Honolulu), leg 6 (13th May 2011, Honolulu-10th June 2011, Cartagena de Indias) and leg 7 (19th June 2011, Cartagena de Indias-14th July 2011, Cartagena). The echosounder was calibrated at the start of the expedition and calibration parameters were updated in the data acquisition software (ER60) i.e., the logged raw data are calibrated. We also provide a data summary of the acoustic data in the form of post-processed products.

## Abstract (summary)

**Table:** 

Measurement(s)	sound speed
Technology Type(s)	echo sounder
Factor Type(s)	time • depth • latitude • longitude
Sample Characteristic - Organism	Metazoa
Sample Characteristic - Environment	ocean biome • tropical • subtropical • temperate
Sample Characteristic - Location	global

Machine-accessible metadata file describing the reported data: 10.6084/m9.figshare.15097260

## Background & Summary

In the frame of fisheries assessment, echosounder data are routinely collected around the world^1,2^, and acoustic data are being used increasingly to study several different features of aquatic ecosystems^3–6^. However, there is still a disconnect between biological oceanography and fisheries research, and often cruises that sample the deep ocean (i.e., the mesopelagic zone from 200 to 1,000 m and beyond) do not collect echosounder data even if the instruments are available onboard.

The 2010 Malaspina circumnavigation expedition aimed at studying the biodiversity and the impact of global change on the deep ocean. The interdisciplinary project collected samples and data across multiple disciplines - from physics and chemistry to genomics and biodiversity^7^ - producing a number of new insights about the ocean^8–13^. Echosounder data collected during the Malaspina expedition have resulted in numerous publications related to mesopelagic fish biomass and behavior^8,14–16^. However, the data still contain a huge amount of information across a range of spatial (meters to oceans basin) and temporal (seconds to seasonal trends) scales that could be exploited in different ways: the objective of this paper is to provide adequate access to those data.

## Methods

**Fig. 1:**
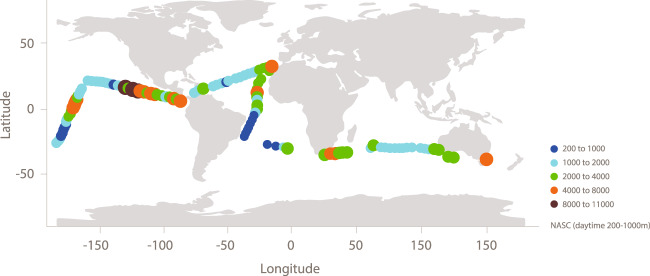
Cruise track and integrated backscatter at different stations (NASC, daytime 200 to 1000 m).

Figure 1 presents the track of the eight-month cruise, and Table 1 provides the detail of the legs and dates. On a routine basis R/V *Hesperides* sailed at an average speed of 11 knots from around 3 pm to 4 am (local time). The vessel arrived on station at around 4 am daily to carry out sampling operations at a fixed point for about 11 hours.

**Table 1:** Dates and starting points of the 7 legs of the Malaspina cruise.

	Start date and location	End date and location
Leg1	14th December 2010, Cádiz	13th January 2011, Rio de Janeiro
Leg2	17th January 2011, Rio de Janeiro	6th February 2011, Cape Town
Leg3	11th February 2011, Cape Town	13th March 2011, Perth
Leg4	17th March 2011, Perth	30th March 2011, Sydney
Leg5	16th April 2011, Auckland	8th May 2011, Honolulu
Leg6	13th May 2011, Honolulu	10th June 2011, Cartagena de Indias
Leg7	19th June 2011, Cartagena de Indias	14th July 2011, Cartagena

Acoustic measurements were carried out continuously using a Simrad EK60 echosounder), operating at 38 and 120 kHz (7° beamwidth transducers) with a ping rate of 0.5 Hz. Unfortunately, the 120 kHz failed during the first leg of the cruise and only 38 kHz data were collected. Echosounder observations were recorded down to 1000 m depth. The echosounder files are in the proprietary Simrad raw format and can be read by various softwares (e.g., LSSS, Echoview, Sonar5, MATECHO, ESP3, echopype, pyEcholab). GPS locations and calibration constants are imbedded in each file.

Additionally, daytime data integrated over 2 m vertical bins from 200 to 1000 m depth are provided as Nautical Area Scattering Coefficient (NASC). Each “voxel” is the average of all cleaned and validated data recorded over that depth range, in a time period starting 8 hours before the start of the station (defined as start of the CTD cast) and ending 8 hours after the start of the station, with only data recorded in the period between 1 hour after local sunrise and 1 hour prior to local sunset accepted (i.e., during local daytime hours, but removing crepuscular periods when vertical migration of biota is strong). The relatively long interval over which data were accepted around each station was chosen since the station sampling resulted in noisy acoustic data,, a long interval was therefore chosen to ensure valid data on all stations.

Finally, summaries of per station daytime and nighttime acoustic data (omitting data recorded within 1 hour of sunrise and sunset) are provided. The data fields in this file are station date, latitude and longitude, and per day and night average NASC 200–1000 m, average NASC 0–1000, weighted mean depth (WMD) of NASC 200–1000 m, migration amplitude, NASC day-to-night ratio and migration ratio.

## Data Records

The raw data are available at PANGAEA (10.1594/PANGAEA.921760^17^). The daytime data integrated over 2 m vertical bins are available at (10.1594/PANGAEA.923087^18^) and the summary file is available at (10.1594/PANGAEA.926619^19^).

## Technical Validation

The echosounder was calibrated before departure (30th of November 2010, close to Mazarron port, water temperature ca. 17 °C) and values of the peak transducer gain (G_0_) and Simrad correction factor (S_a_) for both frequencies were updated in the ER60 software following the standard target method^20^. Briefly, a 38.1 mm diameter tungsten carbide sphere was positioned under the vessel at a range of 12 m using nylon cable, and moved systematically through the acoustic beams of both transducers (38 and 120 kHz). The ER60 software compares measured values of the sphere’s target strength at each position in the beam with model predicted values, and estimates the transducers’ calibration parameters (G_0_, S_a_ and beam widths) for the given operational settings (see Table 2). Data are stored in raw proprietary Simrad format (.raw,.idx,.bot files).

**Table 2:** Calibration settings and results.

Frequency (kHz)	Pulse length (ms)	Power (W)	G_0_ (dB re 1)	S_a_ (dB re 1 m^−1^)	EBA (dB re 1 Steradian)
38	1.024	1600	24.05	−0.62	−20.6
120	1.024	250	25.45	−0.37	−21

Values of peak transducer gain (G_0_) and the Simrad correction factor (S_a_) were applied prior to the expedition.

## Usage Notes

**Fig. 2:**
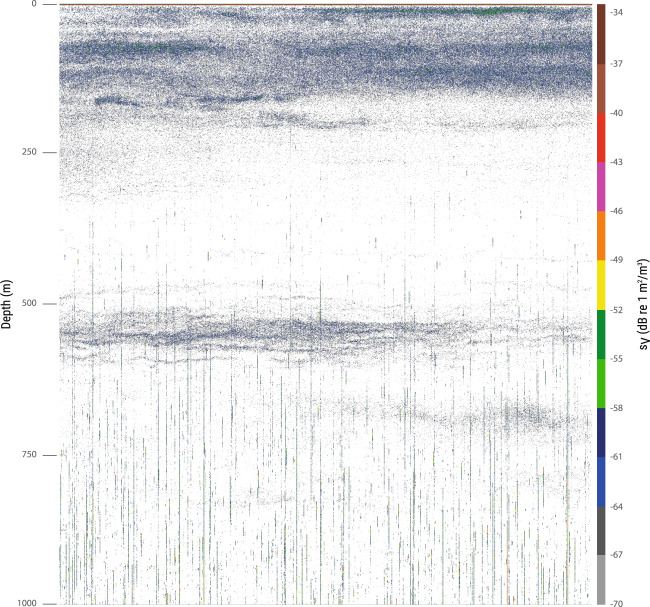
Example of echogram affected by noise (vertical spikes) for average vessel speed over 11 kn in Leg 2 (18/01/2011 22:06–22:46 h).

**Fig. 3:**
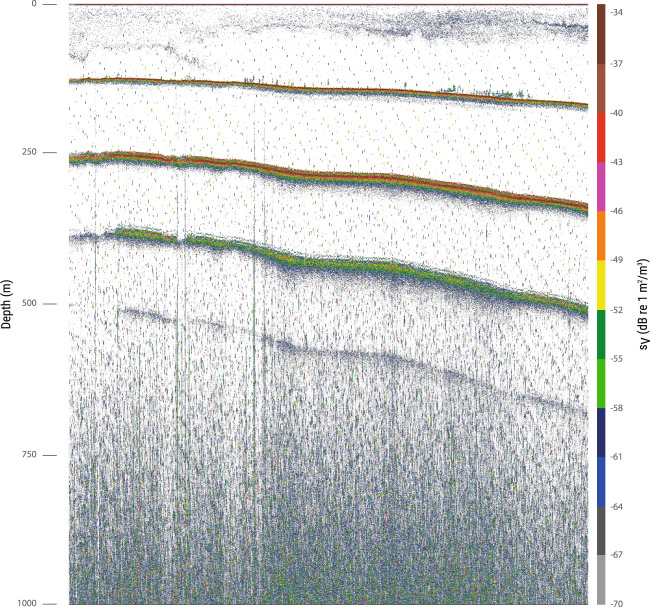
Example of echogram affected by interference from other acoustic equipment near the coast, in shallow waters, during Leg 4 (11/04/2011 21:26–22:04 h).

The main objective of this paper is to allow researchers full access to the data so they can analyze them with objectives that might be completely different from the ones they were collected for. Therefore, the raw data are provided so that each researcher may analyze them using the appropriate methodologies that will differ from those used in previous studies. However, we recommend paying attention to some of the specificities of the cruise that are important when filtering the data. In particular and depending on the objectives data should be filtered for noise following available techniques^21–23^. During the Malaspina expedition, on-station sampling took place from early morning to the beginning of the afternoon, whereas underway navigation occurred from the afternoon and through the night. Therefore, most of the daytime echo sounder data correspond to a stationary situation, and the level of noise is different from day to night (see materials and methods in Irigoien *et al*., 2014). The data were subjected to different types of noise along the survey. The most common noise was caused by the propeller and/or wave-hull collisions occurring at vessel speeds over 11 knots and were seen as long spikes on the echogram (example in Figs. 2 and 3, see also supplementary figure 7 in Irigoien *et al*.^8^ for filtering results). It is recommended to treat these data, especially for depths deeper than 100–200 m, using appropriate filters^24^. On some occasions, data collected near the coast, in shallow waters, presented interference from other acoustic equipment onboard. This was seen as typical short “flecks” present in pings separated by constant intervals, gradually increasing its range of appearance on the echogram. This type of noise can also be removed by means of impulsive noise filters^24^. Some parts of the data were also affected by bad weather conditions, this typically seen on the echogram as backscattering losses in some consecutive pings. This can also be treated with well-established filters as the attenuated signal removal. Another issue to be highlighted is that, due to technical problems in some small parts of the tracks, the 38 kHz frequency was in passive mode. Our previous work being focused in the mesopelagic, the 120 kHz data were not used. A preliminary analysis of the 120 kHz data showed to have a high noise to signal ratio. We have included them here as it is not possible to evaluate if useful for specific objectives or using different filtering approaches, however caution is recommended if using the 120 kHz data. Finally, 120 kHz data were not recorded during the first leg due to malfunctions of the transponder.

## References

[1] Planque B, Vaz S (2011). Understanding what controls the spatial distribution of fish populations using a multi-model approach. Fish. Oceanogr..

[2] Horne, J. K. & Jech, J. M. Models, measures, and visualizations of fish backscatter. in *Sounds in the sea: From ocean acoustics to acoustical oceanography* (ed. Medwin, H.) 374–397 (Cambridge Univ. Press., 2005).

[3] Eyring CF, Christensen RJ, Raitt RW (1948). Reverberation in the Sea. J. Acoust. Soc. Am..

[4] Koslow, J. A. The role of acoustics in ecosystem-based fishery management. *ICES J. Mar. Sci. J. du Cons*. fsp082 (2009).

[5] Lezama-Ochoa A (2011). Spatial patterns and scale-dependent relationships between macrozooplankton and fish in the Bay of Biscay: an acoustic study. Mar. Ecol. Ser..

[6] Ariza A, Garijo JC, Landeira JM, Bordes F (2015). Progress in Oceanography Migrant biomass and respiratory carbon flux by zooplankton and micronekton in the subtropical northeast Atlantic Ocean (Canary Islands). Prog. Oceanogr..

[7] Duarte CM (2015). Seafaring in the 21st century: the Malaspina 2010 circumnavigation expedition. Limnol. Oceanogr. Bull..

[8] Irigoien, X. *et al*. Large mesopelagic fishes biomass and trophic efficiency in the open ocean. *Nat. Commun*. **5** (2014). 10.1038/ncomms4271 PMC3926006 24509953

[9] Cózar, A. *et al*. Plastic debris in the open ocean. *Proc. Natl. Acad. Sci. USA* **111** (2014). 10.1073/pnas.1314705111 PMC4104848 24982135

[10] de Puelles, M. L. F. *et al*. Zooplankton abundance and diversity in the tropical and subtropical ocean. *Diversity* **11** (2019).

[11] Villarino, E. *et al*. Large-scale ocean connectivity and planktonic body size. *Nat. Commun*. **9** (2018). 10.1038/s41467-017-02535-8 PMC5762663 29321528

[12] Hernández-León S (2020). Large deep-sea zooplankton biomass mirrors primary production in the global ocean. Nat. Commun..

[13] Duarte CM (2020). Sequencing effort dictates gene discovery in marine microbial metagenomes. Environ. Microbiol..

[14] Klevjer, T. A. *et al*. Large scale patterns in vertical distribution and behaviour of mesopelagic scattering layers. *Sci. Rep*. **6** (2016). 10.1038/srep19873 PMC4728495 26813333

[15] Prihartato, P. K., Irigoien, X., Genton, M. G. & Kaartvedt, S. Global effects of moon phase on nocturnal acoustic scattering layers. *Mar. Ecol. Prog. Ser*. **544** (2016).

[16] Aksnes DL (2017). Light penetration structures the deep acoustic scattering layers in the global ocean. Sci. Adv..

[17] Martinez U (2020). PANGAEA.

[18] Klevjer TA (2020). PANGAEA.

[19] Klevjer TA (2021). PANGAEA.

[20] Demer, D. A. *et al*. Calibration of acoustic instruments. *ICES Cooperative Research Report No 326*10.25607/OBP-185 (2015).

[21] Saunders RA (2015). Predatory impact of the myctophid fish community on zooplankton in the Scotia Sea (Southern Ocean). Mar. Ecol. Prog. Ser..

[22] Watkins JL, Brierley AS (1996). A post-processing technique to remove background noise from echo integration data. ICES J. Mar. Sci..

[23] Socha DG, Watkins JL, Brierley AS (1996). A visualization-based post-processing system for analysis of acoustic data. ICES J. Mar. Sci..

[24] Ryan TE, Downie RA, Kloser RJ, Keith G (2015). Reducing bias due to noise and attenuation in open-ocean echo integration data. ICES J. Mar. Sci..

